# Sequential Event Rate Monitoring

**DOI:** 10.1002/sim.70359

**Published:** 2026-01-22

**Authors:** Dong‐Yun Kim, Sung‐Min Han

**Affiliations:** ^1^ National Institutes of Health Bethesda Maryland USA; ^2^ i2KIE, LLC Gaithersburg Maryland USA

**Keywords:** clinical trial, continuous event rate monitoring, sequential probability ratio test, staggered entry, time‐to‐event‐data

## Abstract

Effective monitoring of event rates is essential for maintaining statistical power and study integrity in clinical trials, particularly when the primary endpoint involves time‐to‐event outcomes. We propose Sequential Event Rate Monitoring (SERM), a new and innovative approach for continuous monitoring of event rates. SERM leverages the Sequential Probability Ratio Test (SPRT) with improved boundaries derived from the nonlinear renewal theorem by Kim and Woodroofe (2003). This method represents the first practical implementation of their theoretical work in this area. SERM offers several tangible benefits, including ease of implementation, efficient use of data, and broad applicability to trials. Decision boundaries can be directly obtained from simple formula. A detailed illustration of the method using real‐world data from a large Phase III clinical trial demonstrates its potential for rapid assessment. SERM operates on blinded data so it can be used in tandem with a broad range of study designs while preserving study integrity. Although slow patient accrual lengthens the time needed to reach a conclusion, it does not significantly affect type I or type II errors associated with the decision. This new method provides a robust tool for enhancing trial monitoring, enabling timely and informed decision‐making in diverse clinical settings.

## 1 Introduction

During the course of a trial, key quantities of interest such as patient accrual, the number of primary events, and adherence rates are routinely included in periodic Data Coordinating Center reports and discussed in an open session of a Data and Safety Monitoring Board meeting. Also, for a placebo‐controlled randomized clinical trial, such reports are usually based on blinded data to preserve the integrity of the study design. So, for the number of primary events, only the combined count from both placebo and treatment arms is reported.

The number of events as a primary outcome, especially in time‐to‐event data, is of particular interest to stakeholders because it is directly related to statistical power. To be able to detect an effect size with high probability, say 85% or more, the investigators design a study so that the minimum number of primary events is realized by the end of the study.

However, the number of primary events alone is inadequate to assess the rate of primary events if it involves time‐to‐event data, particularly during the early phase of the trial. During active recruitment, due to the staggered nature of patient entry, the vast majority of events are administratively censored at any given point. A better approach is to reformulate the problem in terms of the event rate, rather than event count. This is because the event rate approach not only takes the count of events into consideration, but also accounts for the accumulated times in between events as well, making more efficient use of information from data.

Informal event monitoring has been employed for many years in numerous clinical trials, and has been quite successful in many cases. Unfortunately, the ability to accurately assess the results relies heavily on the vast experience and skillful interpretation of the investigator. With many different things going on at the same time, the investigator might miss the opportunity to “correct the ship” in time.

We believe that a more formal approach, using a statistical test in event rate monitoring, takes much of the guesswork and uncertainty out of it and significantly lessens the burden on the investigator.

In this article, we propose Sequential Event Rate Monitoring (SERM), a new, continuous monitoring method for the event rate for time‐to‐event data in a clinical trial. This method is the first practical implementation of the work by Kim and Woodroofe [1]. They observed that the Sequential Probability Ratio Test (SPRT) for the parameter of a survival distribution under staggered entry and censoring can be approximated by a perturbed random walk. They also proved a nonlinear renewal theorem that provides the theoretical basis for obtaining the sequential boundaries given type I and type II errors.

Since its introduction in 1947 [2], SPRT has been successfully used in many different areas. This owes to the fact that SPRT minimizes the expected sample size for given type I and type II errors under simple hypotheses. Unlike a fixed sample hypothesis test, however, SPRT has two decision boundaries. The test supports the alternative hypothesis if the test statistic up‐crosses the upper boundary first; conversely, it supports the null hypothesis if the test statistic down‐crosses the lower boundary first; if neither boundary is crossed, more data are collected until a decision is reached. With probability one, the test reaches a decision with a finite sample.

In many applications, the sequential boundaries suggested by Wald have been used for their simplicity and universality. On the other hand, his boundaries are conservative in general, resulting in a wider interval than necessary to keep type I and type II error under control. Because of this, it takes longer to reach a decision with a potential for wasted time, cost, and opportunity; this could be substantial in large‐scale clinical trials or in studies where the data are scarce.

In two articles [3, 4], Lai and Siegmund showed that SPRT can be approximated by a perturbed random walk, and the sequential boundaries from renewal theorems tend to be narrower than Wald's. However, they considered only complete cases so their results are not suitable for time‐to‐event data where censoring is the norm, not the exception, as in most clinical trials.

On the other hand, SERM can handle continuous monitoring by virtue of SPRT and the results by Kim and Woodroofe because their asymptotic theory is applicable to time‐to‐event data in a clinical trial.

There are some useful features of the method. First, the method works with blind data, so the monitoring can be performed independent of the study design, while preserving study integrity. Second, slow patient accrual does not increase the chance of making a faulty decision, although it may take longer to reach a boundary under such circumstances. Given the fact that numerous trials suffer from slow accrual, this is a key advantage of the proposed method. Third, sequential boundaries corresponding to a wide range of design parameters can be calculated from a set of simple trigonometric functions without requiring lengthy simulations.

By design, the monitoring statistic is updated frequently, i.e., at each new patient addition during the active recruitment phase, or at regular intervals during follow‐up. Therefore, the method works best in a centralized data management system. Given such a system, the method can be easily incorporated into dashboard‐type applications.

There are other sequential monitoring approaches. Kulldorff et al. [5] address safety monitoring using the maximized sequential probability ratio test (MaxSPRT) under a composite hypothesis, where the decision boundary is determined via simulation. Their method is developed for count data following Poisson or binomial distributions—not for time‐to‐event data, which involves censoring and is typically more complex to handle.

Silva et al. [6] provide practical examples for designing and conducting sequential hypothesis testing and introduce several sequential methods. However, as in [5], these methods are based on binary and Poisson data, rather than time‐to‐event data such as that used by SERM.

On the other hand, Martens et al. [7] are applicable to time‐to‐event data. Their approach assumes each patient has the same fixed length of observation period, which is more likely short‐term in nature. In SERM, varying follow‐up times are built into the model. Moreover, their approach is focused on type I error control, while SERM controls both type I and II errors, as derived from the nonlinear renewal theory.

This article is organized as follows. In Section 2, we introduce key notations and explain how to set up sequential boundaries given a set of design parameters. In this section, we also discuss the result of simulations for the average stopping time in terms of both the time period and the number of primary events for selected combinations of accrual size, length of recruitment period, and minimum follow‐up time.

In Section 3, we illustrate the method using a well‐known large Phase III placebo‐controlled randomized trial. In the last section, we mention additional features, limitations, and potential applications of the method in other areas.

## 2 Sequential Event Rate Monitoring (SERM)

### 2.1 Setup

Consider a clinical trial where the time‐to‐event is of primary research interest. Assume that patients enter the trial in a staggered fashion, and entry times $0=\tau_{0}<\tau_{1}<\cdots$ form a Poisson process with known intensity λ>0.

Let $Y_{i}$ denote the time from entry to event of the *i*th patient, i=1,…, and assume that $Y_{i}$ follows an i.i.d. exponential distribution with unknown rate θ, that is, $E\left(Y_{i}\right)=1/\theta .$ Let N(t) denote the number of patients who have entered the clinical trial by time t>0, that is, $N\left(t\right)=\inf\left\{i;\tau_{i}>t\right\}$.

Let $\left[0,T_{0}\right]$ and $T\left(>T_{0}\right)$ denote the recruitment period and the end of follow‐up time, respectively. Let $n_{0}$ denote the planned accrual size. Conditional on $N\left(T_{0}\right)-1=n_{0},$ $\left\{\tau_{1},\ldots ,\tau_{n_{0}}\right\}$ forms a random sample from the uniform distribution on $\left[0,T_{0}\right].$ Consequently, the average entering time of patients is $T_{0}/2$. Therefore, the average follow‐up time for patients is $T_{a}=T-T_{0}/2$, assuming there is no loss to follow‐up.

Let p denote the (unknown) proportion of patients whose lifetime after entry is at most $T_{a}$. We wish to test $H_{0}:p=p_{0}$ versus $H_{1}:p=\delta p_{0}$ for some $0<p_{0}<1$ and a design parameter δ>0. Without loss of generality, we assume 0<δ<1 throughout the article.

By a simple transformation $\theta_{j}=-T_{a}^{-1}\ln\left(1-\delta^{j}p_{0}\right)$ for j=0,1, the hypotheses are equivalent to $H_{0}:\theta =\theta_{0}\;\mathrm{vs}.H_{1}:\theta =\theta_{1}.$ By rescaling the patient entering time and the survival time with $Y_{i}^{*}=\theta_{0}Y_{i}$ and $\tau_{i}^{*}=\theta_{0}\cdot \tau_{i},$ respectively, the hypotheses are reduced to $H_{0}:\theta^{*}=1\;\mathrm{vs}.H_{1}:\theta^{*}=\delta^{*}$ where $\delta^{*}=\ln\left(1-\delta p_{0}\right)/\ln\left(1-p_{0}\right)$.

The log‐likelihood ratio test statistic at time $\tau_{n}^{*}$ is of the form

(1) $$\Lambda_{n}=K_{n}\ln\left(\delta^{*}\right)+\left(1-\delta^{*}\right)T_{n}$$

where $K_{n}=\sum_{k=1}^{n}1_{\left\{Y_{k}^{*}\leq \tau_{n}^{*}-\tau_{k-1}^{*}\right\}}$ and $T_{n}=\sum_{k=1}^{n}\min\left(Y_{k}^{*},\tau_{n}^{*}-\tau_{k-1}^{*}\right)$; $1_{A}$ denotes the indicator function of event A. Thus, $K_{n}$ is the number of events and $T_{n}$ is the total‐time‐on‐test by $\tau_{n}^{*}$, for n=1,2,….

Let $N_{a}=\inf\left\{n\geq 1:\Lambda_{n}>a\right\}$ and $M_{b}=\inf\left\{n\geq 1:\Lambda_{n}<-b\right\}$ for some fixed positive constants a,b. The test statistic $\Lambda_{n}$ is updated each time a new patient enters the trial until the statistic crosses either a or −b, that is, until $\left\{\Lambda_{n}>a\right\}$ or $\left\{\Lambda_{n}<-b\right\}$ occurs. The test rejects the null hypothesis if and only if $N_{a}<M_{b}$. On the other hand, it accepts the null hypothesis if and only if $M_{b}<N_{a}$. See Kim and Woodroofe [1] for details.

In the case that boundary crossing does not occur by the time the last patient enters, the statistic can be periodically updated thereafter, for example, daily, until the end of the study period.

### 2.2 Determination of Sequential Boundaries

Let α,β denote type I and type II errors associated with SPRT, respectively. Then a,b can be written as

(2) $$a=\ln\left(\frac{\left(1-\beta \right)\gamma_{1}}{\alpha }\right),b=\ln\left(\frac{\left(1-\alpha \right)\gamma_{0}}{\beta }\right)$$

where $\gamma_{0},\gamma_{1}$ denote the Laplace transform of the excess limiting distribution under the null and alternative hypotheses, respectively [8].

$\gamma_{0}$ and $\gamma_{1}$ have no closed form in general, so these values need to be numerically obtained via computer simulations. Kim and Woodroofe [1] provided sequential boundaries for a few selected cases, but these values are not adequate to cover the diverse scenarios required for monitoring in a clinical trial. Obviously, there is a need for simple formula that can be used to compute sequential boundaries without simulations.

**FIGURE 1:**
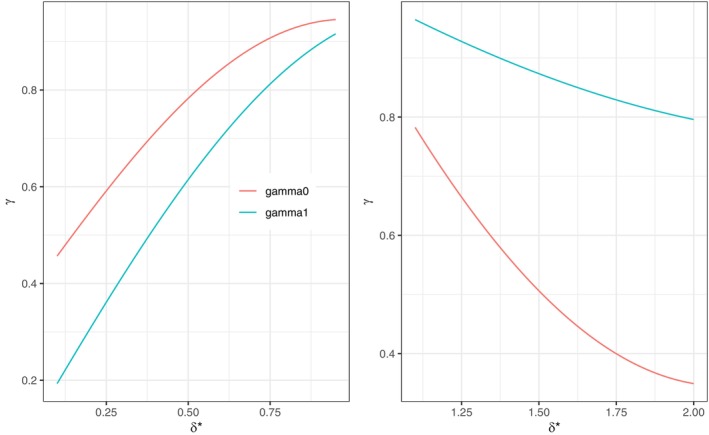
Graphs of $\gamma_{0},\gamma_{1}$.

With this in mind, we conducted computer simulations to determine the functional relationship between $\delta^{*}$ and $\gamma_{j},j=0,1$. The range [0.2,0.8]∪[1.2,2.0] for $\delta^{*}$ was divided into grids of size 0.01. At each grid point within the range, 500 000 simulations were performed to evaluate $\gamma_{j}$. Then nonlinear regression was used to describe the relationship between $\delta^{*}$ and $\gamma_{j}$. We found trigonometric functions of the form $\gamma_{j}=a_{1}\sin\left(a_{2}\;\delta^{*}\right)+a_{3}$ for j=0,1 to be satisfactory for the purpose. Coefficients $a_{i},i=1,2,3$ depend on the range of $\delta^{*}$ (Table 1). Figure 1 shows the graphs of $\gamma_{j}$ as a function of $\delta^{*}$.

**TABLE 1:** Coefficients for $\gamma_{0},\gamma_{1}$.

$\delta^{*}$	$\gamma_{j}$	$a_{1}$	$a_{2}$	$a_{3}$
[0.2,0.8]	$\gamma_{0}$	0.582	1.606	0.364
	$\gamma_{1}$	0.888	1.297	0.078
[1.2,2.0]	$\gamma_{0}$	−1.648	0.750	1.993
	$\gamma_{1}$	−0.534	0.616	1.300

We conducted additional simulations using the approximation functions at several input points across the range. To evaluate the performance of these functions, we performed computer simulations to compare the nominal versus empirical type I and type II errors under selected δ values ranging from 0.30 to 0.8. We investigated two situations: α=0.05,β=0.1, and α=β=0.1. In each case, an exponential distribution of rate 1 or δ was used to generate lifetime data, depending on whether we were assessing type I or type II errors. Each scenario was replicated 10 000 times, and the summary of the simulations is shown in Table S1. For corresponding simulations where δ values ranged between 1.1 and 1.5, refer to Table S2.

In each case, empirical type I and type II errors were reasonably close to their nominal values, although there was a tendency for type II errors to slightly increase for larger values of δ>1. Overall, we are confident that the approximation captures the essence of their relationship reasonably well, and that it could be used in event rate monitoring.

The original formulation of the sequential boundaries assumes that the exponential lifetime distribution is not randomly censored. For practical applications in clinical trials, censoring occurs for various reasons, for example, loss to follow‐up or dropouts. To evaluate how the likelihood ratio test performs under random censoring, we used an independent exponential variable to randomly censor the exponential lifetime data. The rate parameter for the censoring variable was determined by the censoring proportion, and the rate parameter for the lifetime distribution. We chose 0.2 for the censoring proportion, a common number used in practice, and generated censored data with the censoring indicator.

To accommodate the censoring information, we modified the likelihood ratio test such that while the censored individual contributes to $T_{n}$ as usual until the point of censoring, the individual does not add to the event count $K_{n}$. For each setup, we used α=0.05 and β=0.1 for nominal type I and type II errors. A range of δ values from 0.3 to 0.8, and 1.2 to 1.5 were used and empirical type I and type II errors were estimated.

Overall empirical type I errors for δ<1 are comparable to the uncensored cases. Type II errors varied from 0.899 to 0.924 in this range. On the other hand, for δ>1 cases, empirical type I errors were smaller than nominal 5%, ranging from 0.037 to 0.046. Type II errors were slightly larger than nominal 10%, ranging from 0.12 to 0.141 with a slight increasing tendency toward larger δ values.

### 2.3 Average Stopping Time

Since the time until the monitoring scheme raises a signal is a random quantity, the average stopping time is of practical interest for monitoring purposes. Table 2 summarizes the results of computer simulations for various combinations of event rate $p_{0}$, recruitment size $n_{0}$, recruitment period $T_{0}$, and minimum follow‐up period $T_{f}$. We focused on selected combinations of $T_{0}=1$, 2, and 3 years, and $T_{f}=3$, 2, and 1 years, respectively, since these are typical scenarios for large phase III trials.

**TABLE 2:** Average stopping time $E\left(\tau_{N_{1}}\right)$ (unit: days).

$p_{0}$	$n_{0}$	$T_{0}$	$T_{f}$	$E\left(\tau_{N_{1}}\right)$	$\mathrm{Sd}\left(\tau_{N_{1}}\right)$	$E\left(a_{1}\right)$	$\mathrm{Sd}\left(a_{1}\right)$
0.05	4500	365	1095	537	279	50	33
0.05	4500	730	730	748	338	51	34
0.05	4500	1095	365	931	391	50	33
0.10	2200	365	1095	523	276	48	32
0.10	2200	730	730	727	326	48	32
0.10	2200	1095	365	917	384	49	32
0.15	1400	365	1095	513	271	46	30
0.15	1400	730	730	720	327	46	31
0.15	1400	1095	365	907	387	47	31
0.20	1000	365	1095	517	276	45	30
0.20	1000	730	730	712	329	44	30
0.20	1000	1095	365	900	379	44	29
0.25	800	365	1095	486	257	42	28
0.25	800	730	730	683	309	42	28
0.25	800	1095	365	869	369	43	28
0.30	600	365	1095	491	260	39	25
0.30	600	730	730	689	313	40	26
0.30	600	1095	365	877	376	40	26
0.35	500	365	1095	476	255	37	25
0.35	500	730	730	669	306	38	25
0.35	500	1095	365	847	363	38	24
0.40	400	365	1095	474	257	35	23
0.40	400	730	730	669	308	35	23
0.40	400	1095	365	842	367	35	23
0.45	350	365	1095	447	239	33	21
0.45	350	730	730	631	294	33	22
0.45	350	1095	365	816	355	33	22
0.50	300	365	1095	433	234	31	20
0.50	300	730	730	613	289	31	20
0.50	300	1095	365	791	343	31	20

The simulation was conducted as follows. In each scenario, a significance level α=5% and a power of 90% were used. The design parameter δ=0.75 was chosen because it is a commonly used figure in informal monitoring.

1. Set up the hypotheses $H_{0}:p=p_{0}$ versus $H_{1}:p=\delta p_{0}$.
2. Calculate $\delta^{*}$ using the formula in Section 2.1, and determine $\gamma_{0}$ and $\gamma_{1}$ from Table 1. Substitute these values into Equation (2) to find the upper and lower boundaries a and −b.
3. Generate $n_{0}$ patient entry times from uniform $\left[0,T_{0}\right]$ distribution.
4. Using the simple transformation described in Section 2.1, compute $\theta_{0}$.
5. Generate patient survival data from an exponential distribution with hazard rate $\theta_{0}$.
6. Rescale patient entry and survival times as described in Section 2.1.
7. Update the log‐likelihood ratio (LLR) test statistic using Equation (1).
8. While the test statistic falls between a and −b (exclusive), repeat Step 7.

We simulated different values of $n_{0}$ for each $p_{0}$ and identified the minimum $n_{0}$ required to ensure the 99.5% *completion rate*, i.e., the monitoring reaches a decision by the end of the study period at least 99.5% of the time. $\tau_{N_{1}}$ was treated as NA if the test statistic did not cross a boundary by T.

For each combination, the average stopping time $E\left(\tau_{N_{1}}\right)$ (in days) and its standard deviation $\mathrm{Sd}\left(\tau_{N_{1}}\right)$, the average number of events $E\left(a_{1}\right)$ that occurred until stopping time $\tau_{N_{1}}$, and its standard deviation $\mathrm{Sd}\left(a_{1}\right)$ were estimated based on 10 000 simulations under the null distribution. For example, in Table 2, when $p_{0}=0.25$ and $n_{0}=800$, the average number of required events is ˜42.

The table shows that the average number of events clearly has an inverse relationship with event rate $p_{0}$. On the other hand, it is not significantly influenced by either $T_{0}$ or $T_{f}$. This is not true for the average stopping time $E\left(\tau_{N_{1}}\right)$.

We conducted simulations under the alternative, and the results were are quite similar, except that the average stopping time is slightly smaller than what were reported in the table. This reflects the fact that the nominal values for type I and type II errors were 0.05 and 0.1, respectively.

### 2.4 Empirical Type I and Type II Errors

To investigate the empirical type I and type II errors under the clinical trial scenario, we conducted simulations where their nominal counterparts were 0.05 and 0.1, respectively. In each scenario, δ=0.75 was used for an alternative hypothesis, and 5000 repetitions were performed. The results are summarized in Table 3 below. In general, the empirical errors are well aligned with the nominal values under various combinations of event rate ($p_{0}$), sample size ($n_{0}$), recruitment period ($T_{0}$), and minimum follow‐up period ($T_{f}$).

**TABLE 3:** Empirical type I and II errors (time unit: days).

$p_{0}$	$n_{0}$	$T_{0}$	$T_{f}$	TypeI	TypeII
0.05	4500	365	1095	0.060	0.095
0.05	4500	730	730	0.054	0.095
0.05	4500	1095	365	0.064	0.098
0.10	2200	365	1095	0.048	0.101
0.10	2200	730	730	0.053	0.099
0.10	2200	1095	365	0.054	0.100
0.20	1000	365	1095	0.050	0.089
0.20	1000	730	730	0.056	0.093
0.20	1000	1095	365	0.055	0.087
0.30	600	365	1095	0.058	0.092
0.30	600	730	730	0.053	0.097
0.30	600	1095	365	0.052	0.093
0.40	400	365	1095	0.058	0.101
0.40	400	730	730	0.053	0.099
0.40	400	1095	365	0.057	0.095

For the scenarios listed in Table 2, the average empirical type I and type II errors were 0.056 and 0.091, respectively. The empirical type I errors were slightly above the nominal value of 0.05, whereas the empirical type II errors were slightly below the nominal value of 0.10. The corresponding standard deviations were 0.002 and 0.003, respectively. No clear increasing or decreasing trend was observed.

Additionally, we performed simulations to see how SERM performs when the true ratio δ is substantially different from 1.0 or 0.75, for example, 0.5 or 2.0. To do so, we used the same boundaries to test hypotheses $H_{0}:p=p_{0}$ versus $H_{1}:p=\delta p_{0}$ with δ=0.75. When the true δ=0.5, the probability of accepting $H_{1}$ is 0.9996 on average. On the other hand, if the true δ=2.0, the probability of accepting $H_{0}$ was 1.0. In both cases, the average stopping times were substantially smaller, ˜56% of the reported numbers in Table 2. These results imply that when the actual ratio is above or below the specified hypotheses, SERM chooses the closer hypothesis faster.

Simulations were performed for other values of δ, ranging from 0.6 to 0.8; these are the values most likely to be used in the context of event rate monitoring. Various combinations of $p_{0}$ and $n_{0}$ were used to evaluate the overall performance of SERM, and they are summarized in Table S3.

### 2.5 Sensitivity Analysis

SERM assumes that the lifetime has an exponential distribution. In practice, this may or may not be true. To evaluate its performance under slight deviations from the exponential distribution, we used Weibull distributions with shape parameters 0.95, 1.05, and 1.1. The corresponding rate parameters 1.05, 0.95, and 0.977 were determined based on reasonable proximity to an exponential distribution, especially in terms of hazard, so that the distribution could be fairly compared. Each scenario was replicated 10 000 times.

For type I error simulations, data were generated from Weibull distributions and tested using δ parameters from 0.3 to 0.8 for the alternative hypothesis. In general, empirical type I errors were somewhat smaller than the nominal 5% in each case.

For type II error simulations, data were generated from Weibull distributions with different combinations so that the hazard function was reasonably close to the constant hazard δ=0.75. These simulations show that the empirical type II errors were close to the nominal 10%. This provides evidence that SERM performs quite well under small departure from the exponential distribution.

To further evaluate the robustness of SERM, we conducted a sensitivity analysis examining departures from the exponential distribution. We considered Weibull distributions with shape parameters k ranging from 0.5 to 2.0 and selected corresponding scale parameters to preserve comparability. As in the main analysis, we used δ=0.75 for the alternative hypothesis and calibrated the stopping boundaries to achieve nominal type I error of 5% and power of 90%. Results based on 10 000 simulated datasets per scenario are summarized in Table S4.

The empirical type I error was substantially inflated when the shape parameter was well below 1.0. As the shape parameter approached 1.0, the empirical type I error decreased toward the nominal value, and continued to decline for larger shape parameters. Conversely, power increased monotonically with the shape parameter. Collectively, these findings indicate that when the underlying distribution exhibits a small shape parameter (e.g., k=0.5), empirical type I error is inflated and power is attenuated relative to nominal levels, suggesting that SERM may not be suitable for monitoring in such settings.

## 3 Illustration

Studies Of Left Ventricular Dysfunction Prevention (SOLVD‐P) was part of a double‐blind, randomized, placebo‐controlled multi‐center clinical trial where 23 medical centers in the United States, Canada, and Belgium participated. The purpose of the study was to assess the effectiveness of enalapril, an angiotensin‐converting enzyme inhibitor, on reducing mortality among patients with left ventricular dysfunction but without history of congestive heart failure.

The plan for the trial [9] was to recruit 4600 patients in 3 years. A 3‐year all‐cause mortality for the control (placebo) group was estimated at 17%, and the study was designed to have 90% power to detect a 25% reduction in mortality for the treatment group [10]. This gives an estimated 13% mortality for the treatment group. Applying 1:1 allocation between two arms, the expected overall 3‐year event rate for both groups combined was 15%.

To set up the hypothesis for monitoring, we chose the event rate $p_{0}=0.15$ and design parameter δ=0.75. Then $\delta^{*}=0.734$. This number was plugged into the approximating functions using coefficients in the first and second row of Table 1 to obtain $\gamma_{0}$ and $\gamma_{1}$. Using α=0.05 and β=0.1, the upper boundary a=2.67 and the lower boundary −b=−2.15 were obtained from Equation (2).

**FIGURE 2:**
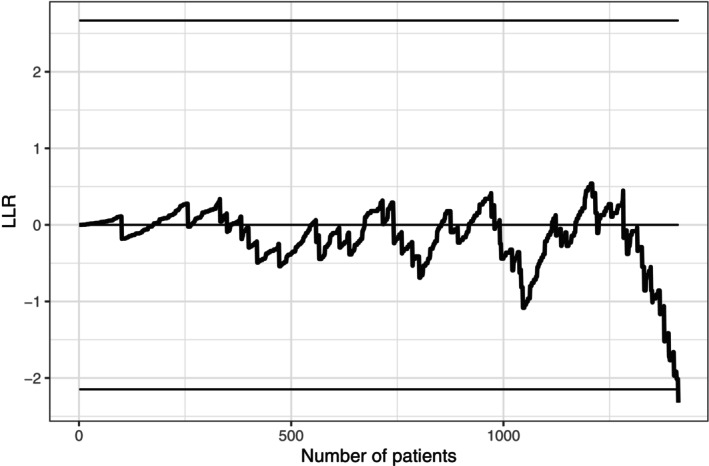
SOLVD‐P monitoring chart.

Raw entry and survival data were transformed as described in Section 2.1. To break ties in patient entry times, small values were randomly added where necessary. The log‐likelihood ratio test statistic in Equation (1) was updated each time a new patient joined the trial. The statistic stopped after down‐crossing the lower bound, as shown in Figure 2.

The boundary crossing occurred around the mid‐point during the recruitment phase, that is, approximately one and a half year after the first randomization. SERM signaled in favor of the expected combined event rate (15%). At this point, about 1410 patients or 31% of the planned accrual joined the trial and 51 primary events occurred. The number of primary events is well in line with the average number of events reported in Table 2, although there is no exact case in the table that matches the study.

Using data accumulated up to that time, a rough point estimate for 3‐year mortality was 15.1%, based on an exponential model. The Kaplan–Meier estimate using the full data set was somewhat lower at 14.4%, but the initial estimate was within 95% CI [13.3%, 15.6%]. The recruitment period was extended by 10 months to compensate for slow recruitment, and the minimum follow‐up time was shortened (from 2 years to 13 months).

Note that it was not possible to give a reliable estimate for 3‐year mortality using the Kaplan–Meier estimator based on available data at the time the monitoring reached the decision.

## 4 Discussion

### 4.1 Recapitulation

In this article, we proposed Sequential Event Rate Monitoring (SERM), a new continuous monitoring method for the event rate where time‐to‐event data are the main focus of a clinical trial. Based on the sequential probability ratio test, SERM proves to be a useful tool to detect departures from the planned rate.

Simulation studies show that, on average, the required number of events to reach one of the decision boundaries is very modest and stable under various combinations of event rate, accrual size, length of recruitment period, and minimum follow‐up time.

The article includes a set of trigonometric functions by which decision boundaries can be obtained without computer simulations.

The method was applied to actual patient data from the SOLVD‐P study, a Phase III, two‐arm, placebo‐controlled randomized clinical trial that evaluated the efficacy of enalapril in reducing overall mortality among patients with left‐ventricular dysfunction. The method reached an early decision during the middle of the active recruitment period when only a few dozen primary events had occurred. The findings by the new monitoring method were supported by conventional analysis using the full data set.

Many clinical trials experience slow patient accrual and a limited number of primary events during the early part of the trial. The fact that the validity of the monitoring decision holds under a low recruitment rate, combined with the modest average number of primary events to reach such decision, demonstrates the potential of this approach.

Since SERM works with blind data and monitors the combined event rate, it can be used in tandem with almost any study design with a sufficiently large sample size.

### 4.2 Interpretation

To put SERM monitoring into practice, it requires careful interpretation. For example, reaching the lower boundary at some point, and thus accepting the null hypothesis, does not say anything about the treatment difference between the placebo and treatment arms. It just indicates that the data collected up to that point give confidence that the study will have sufficient number of primary events, that is, sufficient power to detect the specified effect size at the end of the study. The question of whether the measured treatment difference is statistically significant needs to be answered by the study design itself.

On the other hand, if the monitoring statistic reaches the upper boundary, this could be a strong indication that the study may not have sufficient number of primary events at the end. There are several causes for this “symptom,” so it may call for further investigation. Depending on the circumstances, a corrective action and possible protocol amendment should follow.

### 4.3 Monitoring During Passive Follow‐Up

SERM updates the monitoring statistic each time a new patient enters the trial. This was motivated by the fact that theoretical derivation as well as any updates becomes much more straightforward. If no decision is made during the active recruitment phase, the method continues monitoring on a different update schedule. Since no new patient arrival occurs during the passive follow‐up period, any reasonable regular update schedule will suffice, for example, on daily or weekly basis. If the update schedule is too infrequent, however, it could run the risk of having reached a decision boundary months ago without being aware of it.

### 4.4 Data Quality and Management

In general, data quality is important for any clinical trial, but it is crucial for SERM monitoring. Often, the monitoring method reaches an early decision about the event rate using only a portion of the data, say the first half. This assumes that the trend will continue through the end of the study. If there is a significant change in hazard during the study due to secular trends or if significant heterogeneity exists between early and late participants, the monitoring decision will be less reliable or turn out to be inconclusive at best.

Since SERM requires frequent updates by design, the method works best under a centralized data management system, which is ideally capable of keeping data up‐to‐date at all times. The availability of such system is even more important when multiple sites participate in the trial.

### 4.5 Safety Monitoring

Although we discussed the continuous monitoring method within the context of tracking overall event rate, the method is equally applicable to safety monitoring without much modification. As discussed in the Introduction, there are existing safety monitoring methods. However, [5, 6] were developed for binary or count data, not the time‐to‐event data considered here [7] deals with time‐to‐event data under the assumption of identical follow‐up time. Thus, their scenario is quite different from that of SERM.

To apply SERM to safety monitoring, we suggest choosing $p_{0}$ as the expected proportion of adverse events for the patient population. A value > 1 is set as the design parameter δ and sequential boundaries can be obtained from Table 1. This set up considers time until an adverse event occurs. Proceed as before, updating the monitoring statistic each time a new patient enters the trial, and at regular intervals during the passive follow‐up period.

## Funding

The authors have nothing to report.

## Conflicts of Interest

The authors declare no conflicts of interest.

## Supporting information


**Table S1:** Empirical type I and type II errors for 0.3 < delta < 0.8.
**Table S2:** Empirical type I and type II errors for 1.1 < 1.5.
**Table S3:** Empirical error probabilities in clinical trial setting.
**Table S4:** Sensitivity analysis using Weibull distribution.

## Data Availability

The data that support the findings of this study are openly available in BioLINCC at https://biolincc.nhlbi.nih.gov/home/.
