# Supplementary material for: Sequential Event Rate Monitoring
Source: Stat Med. 2026 Jan 22;45(1-2):e70359. doi: 10.1002/sim.70359 (PMC12828109; doi:10.1002/sim.70359)
Supplement: Supplementary file 1 — Table S1: Empirical type I and type II errors for 0.3 < delta < 0.8. Table S2: Empirical type I and type II errors for 1.1 < 1.5. Table S3: Empirical error probabilities in clinical trial setting. Table S4: Sensitivity analysis using Weibull distribution. [file SIM-45-0-s001.pdf]

Supplementary Tables

Table S1 Empirical Type I and Type II Errors for 0.3< delta <0.8

nominal	0.05	0.90	0.10	0.10
delta	Type1.error	Power	Type I error	Power
0.30	0.049	0.905	0.118	0.912
0.35	0.051	0.905	0.120	0.908
0.40	0.050	0.903	0.119	0.914
0.45	0.046	0.910	0.095	0.907
0.50	0.050	0.091	0.094	0.907
0.55	0.044	0.904	0.093	0.912
0.60	0.043	0.904	0.087	0.915
0.65	0.046	0.906	0.090	0.910
0.70	0.045	0.908	0.088	0.907
0.75	0.047	0.091	0.086	0.907
0.80	0.043	0.904	0.091	0.909

Note. Replication 10,000

Table S2 Empirical Type I and Type II Errors for 1.1 <delta <1.5

nominal	0.05	0.90	0.10	0.10
delta	Type1.error	power	Type1.error	power
1.10	0.046	0.883	0.087	0.891
1.15	0.044	0.884	0.087	0.894
1.20	0.043	0.879	0.089	0.887
1.25	0.046	0.876	0.092	0.887
1.30	0.047	0.872	0.088	0.879
1.35	0.043	0.867	0.089	0.878
1.40	0.047	0.872	0.092	0.873
1.45	0.043	0.862	0.083	0.872
1.50	0.042	0.859	0.082	0.863

Note. Replication 10,000

Table S3 Empirical Error Probabilities in Clinical Trial Setting

	p0=0.05 n0=4500		p0=0.1 n0=2200		p0=0.2 n0=1000		p0=0.3 n0=600	
delta	Type1 error	Power	Type1 error	Power	Type1 erro	Power	Type1 error	Power
0.60	0.061	0.910	0.067	0.906	0.062	0.906	0.068	0.908
0.62	0.068	0.906	0.063	0.909	0.064	0.901	0.063	0.911
0.64	0.059	0.902	0.060	0.905	0.055	0.908	0.065	0.912
0.66	0.060	0.903	0.062	0.905	0.059	0.906	0.061	0.909
0.68	0.060	0.907	0.062	0.911	0.061	0.908	0.063	0.906
0.70	0.062	0.903	0.055	0.910	0.055	0.911	0.057	0.920
0.72	0.057	0.908	0.519	0.904	0.049	0.907	0.060	0.912
0.74	0.056	0.915	0.059	0.907	0.059	0.915	0.059	0.902
0.76	0.056	0.908	0.052	0.908	0.057	0.902	0.056	0.901
0.78	0.054	0.910	0.054	0.897	0.056	0.909	0.054	0.905
0.80	0.057	0.896	0.051	0.903	0.051	0.911	0.052	0.909

Note. T0=Tf=730; replication 10,000

Table S4 Sensitivity Analysis using Weibull Distribution

k (shape)	λ(scale), H0	Type I	λ(scale), H1	Power
0.50	0.25	0.20	0.33	0.53
0.70	0.61	0.13	0.81	0.72
0.90	0.89	0.07	1.19	0.86
1.10	1.09	0.03	1.45	0.94
1.30	1.22	0.01	1.63	0.98
1.50	1.31	0.00	1.75	0.99
2.00	1.41	0.00	1.88	1.00
